# Quantitative Measurement of Adult Human Larynx post General Anesthesia with Intubation

**DOI:** 10.7150/ijms.69425

**Published:** 2022-02-07

**Authors:** Chung Feng Jeffrey Kuo, Jagadish Barman, Shao-Cheng Liu

**Affiliations:** 1 Department of Materials Science & Engineering, National Taiwan University of Science and Technology, Taipei, Taiwan, Republic of China.; 2 Department of Otolaryngology-Head and Neck Surgery Tri-Service General Hospital, National Defense Medical Center Taipei, Taiwan, Republic of China.

**Keywords:** quantitative laryngoscopy, computer-aided diagnostic system, voice, general anesthesia, intubation, sore throat

## Abstract

**Introduction:** Post-anaesthetic sore throat (PAST) is a well-recognized consequence of tracheal intubation; however, quantitative morphometric measurements remain challenging. This study aimed to introduce a special laser projection device that can facilitate computer-assisted, digitalized analysis and provide important information on laryngeal mucosa change, pre and post-surgery under general anesthesia with intubation.

**Materials and methods:** The laryngeal images were captured and divided into the control group and the intubation group. Image processing techniques were used to quantify the post-extubation laryngeal variation, with its distinct color space and texture features. Meanwhile, the maximum length of the vocal fold, vocal width at the midpoint, and maximum cross-sectional area of the glottic space were determined and calculated. These parameters were analyzed and compared pre and post-surgery.

**Results:** A total of 69 subjects were enrolled in this study, comprising 32 subjects in the healthy group and 37 subjects in the intubation group. The color space and texture analysis with contrast and correlation profiles all shows trend toward higher measures in the intubation group than in the healthy group, with statistical significance and outstanding discrimination ability, especially in the interarytenoid region. The incidence of PAST was approximately 46% (17 patients). The gender difference, type of surgery, and the fixation position of the tube were not significantly related to the PAST occurrence. All the eigenvalues showed significant differences pre and post-surgery in the interarytenoid region and a significant trend toward red and increased contrast texture profiles was revealed. Furthermore, the glottic area showed a significant decrease of 25.29%, while the vocal width showed a significant increase post extubation.

**Conclusion:** Our equipment and processing can measure subtle laryngeal changes that would allow a clinician to diagnose postoperative laryngeal inflammation in a simpler and less invasive way. The trend toward red, the increased contrast texture and vocal width, and the reduced glottic space were all compatible with post-intubation inflammatory response, especially in the interarytenoid region. This is important to know so that one can take appropriate steps to alleviate PAST in the future.

## Introduction

Post-anaesthetic sore throat (PAST), a clinical sign of laryngeal injury, is a well-recognized consequence of tracheal intubation with a distressingly high incidence (6.6-90%) 1. Although it appears to be an inflammatory process 2, the etiology of PAST is not clearly understood and it can be caused by other factors, such as infection, reflux esophagitis, smoking, and the use of oropharyngeal airways 3. One of the problems in evaluating PAST is the lack of an objective diagnostic method that is both convenient and effective. There is no consensus in the literature today as to how or when it should be measured. Patients with PAST usually consult an otorhinolaryngologist, and laryngoscopy is the most common diagnostic method. However, traditional laryngoscopy is subjective with low efficacy. PAST remains a significant cause for discomfort and a lessening of the symptoms is worth striving for.

An alternative method of detecting laryngeal pathologies would be to observe and record the change in physical appearance, particularly hue, texture, and target size. The texture could be broken down into subpatterns of color, size, and shape, which give rise to perceived density, coarseness, fineness, and smoothness 4. Mucosa color was supposed to be associated with inflammation and the CIELAB system has become the universally accepted colorimetric reference system for quantifying and communicating color. Over the past decades, few studies have been conducted to elucidate the association between inflammation and mucosa color, due to numerous potentially confounding factors, including the light source of endoscope and image capture system. Meanwhile, due to lack of reliable scaling conversion references to learn the corresponding relationship between image pixels and length units, it was very difficult to measure the absolute value of the human larynx by endoscopic images. Only semi-quantitative information could be provided. Until now, there were no studies that attempted to predict postoperative laryngeal inflammation by detailed and spatial information and complex patterns of the human larynx.

In our previous study 5,6, we developed a new laser device that can be quickly clipped onto a rigid endoscope, which can provide a precise measurement of laryngeal structures with a two-point laser. We also found a way to distinguish the laryngeal inflammatory state resulting from laryngo-pharyngeal reflux by using an artificial neural network (ANN) pattern recognition tool, which is more objective than a reflux finding score 6. With this background knowledge, this study was conducted based on actual clinical data with absolute laryngeal measurement, color, and texture, which can be used in the analysis of mucosa change post general anesthesia with intubation. Hence, the primary aim of this study was to confirm whether our device has distinguishable measurements compared with healthy volunteers and patients undergoing general anesthesia with intubation. The secondary aim was to investigate the change of human larynx post extubation and explore the association between inflammation and laryngeal features, to determine whether laryngeal features could be potential predictors for postoperative inflammation. Our measurement with validated equipment can help to develop techniques for reducing adverse laryngeal effects in the future.

## Materials and Methods

### Equipment and human data acquisition

**Figure 1:**
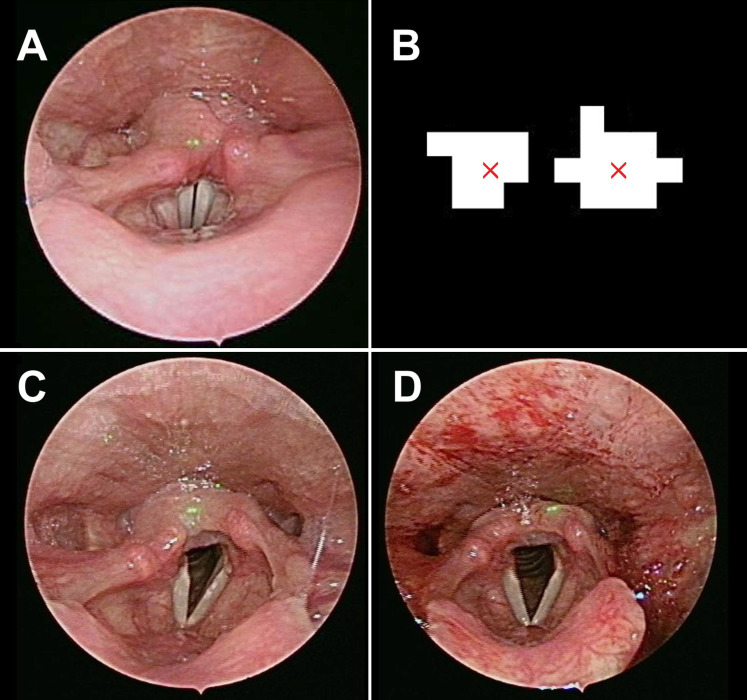
** (A)** The laser beams projected on the healthy control subject. **(B)** Image processing defines the center of the laser dot and gives us its equivalent distance as 1 mm. The laryngeal images pre **(C)** and post **(D)** general anesthesia with intubation.

As in our previous report 5,6, our module comprised a rigid 70° endoscope, laser pointers, and a reflection lens. Two parallel laser beams, with a distance of 1mm, were obtained from a laser excitation apparatus (H435151D/R, 515 nm). The two parallel laser beams can provide the scaling reference for further image analysis (Fig. 1A). In the healthy control group, we collected laryngoscopic images from patients that received any diagnosis other than a laryngeal or hypopharyngeal disease on their visit. The exclusion criteria included recent upper airway disease, history of laryngeal trauma or tumor, smoking, alcoholism, asthmatic patients on inhalation medication, and history of radiotherapy or any kind of neck operations. In the intubation group, the patients must meet the same criteria as those in the healthy control group and they underwent general anesthesia with intubation for surgery, except for lesions of the oral cavity, larynx, or hypopharynx. Laryngoscopy was performed before and within 12-24 hours post-surgery. Our study was not designed for extended follow-up beyond 24 hours, as the process of acute inflammation usually peaks by 24 hours 7. A minimum of 3 evaluations was carried out with 3 cycles of breathing (vocal adduction, glottis opening) and phonation (vocal abduction, glottis closing) during laryngoscopic exam. Thus the observer could acquire and determine the maximum value of laryngeal parameters, including the vocal width, length, area, maximum cross-sectional area of the glottic space, and maximum vocal fold angle, as shown in our previous work 5.

### Image processing

#### Hue feature and CIELab

The videos (1920×1080) were captured and processed by MATLAB. A laryngeal endoscopic image is directly applicable to the RGB (red, green, blue) color space. The RGB color space has rapid calculation and does not need to calculate coordinate conversions. Then, spectroradiometric objective color measurements and the CIELab is used for the variable of laryngeal complexion as it is a color space modeled on the visual pattern of humans and designed to be perceptually uniform in human perceptual work 8. Coordinates of CIELab are L, a, and b respectively, where L* represents the lightness of the color (L* = 0 indicates black and L* = 100 indicates diffuse white), a* represents the position between red and green (negative values means green and positive values means red) and b* represents the position between yellow and blue (negative values means blue and positive values means yellow). The conversion from RGB to CIELab is given by equations (1) and (2), where R, G, and B are pixels values (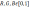) divided by 255 to each pixel value of the image. X, Y, and Z describe the color stimulus, and X_n_, Y_n_ and Z_n_ describe the specific white chromatic reference illuminant.


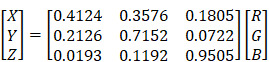

(1)



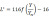





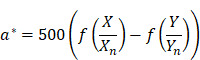




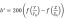

(2)

*Where,*



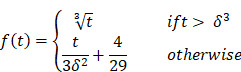



The purpose of this CIE color space is to enhance the color images better than RGB space, where it can represent an infinite number of ranges of chromatic than other color space models. Unlike most of the other objective color measuring methods, CIELab can provide highly accurate non-contact color readings, thus avoiding undesirable edge-loss errors characteristic of contact-type color measuring instruments.

### Textual features

In terms of the perceptual experience of the human eye, the rough and directionality are the primary characteristics used by the human eye to distinguish texture. The Gray-level Co-occurrence Matrix (GLCM) describes the grayness relationship between adjacent pixels in a local area or overall area of an image 9. To quantize the laryngeal variation induced by LPR, this study used the equalization, contrast, correlation, and homogeneity of GLCM to describe the texture information of various regions of the larynx. The angle was set as 0º to analyze the features of LPR. Normalization was performed before the GLCM eigenvalue was extracted and the sum of the elements of GLCM was set as 1 for computing. The eigenvalues used are discussed below.

#### Equalization (*E*)

This eigenvalue is known as the energy, which was used in this study to measure the consistency and equalization of the gray level distribution in each region of an image. Consistency and equalization refer to the probability of the occurrence of a pixel pair and a higher probability of recurrence represents higher consistency and equalization. The range of **(*E*)** of GLCM was [0, 1]. It reaches the maximum value **(*E=1*)** when the gray levels of the image were identical.

#### Contrast (*Con*)

This eigenvalue was used to measure the intensity contrast between adjacent pixels in each region of the image. A larger gray level difference between adjacent pixels represents a larger ***Con*** value of GLCM. In a *k×k* GLCM, the range of ***Con*** would be 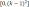.

#### The Correlation (*Cor*)

This one was used to measure the correlation between adjacent pixels in each region.

#### Homogeneity (*Hom*)

It was used to measure the local grayness homogeneity in each region. If the local grayness homogeneity of the image was uniform, the*** Hom*** value would be large.

The definition of our PAST index was calculated as follows:



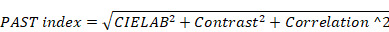



### Statistical analysis

Data were presented as the mean ± standard error of the mean (SEM). All statistical analyses were performed using Student's *t* test or paired samples t-test on GraphPad Prism 5 software (GraphPad Software Inc., La Jolla, CA, USA); *P* < 0.05 was considered statistically significant.

### Ethical considerations

The research protocol (NO: 1-108-05-132) was reviewed and approved by the Institutional Review Board of the Tri-Service General Hospital, Taipei, Taiwan. All methods were performed following the relevant guidelines and regulations. All patients provided written informed consents before participation.

## Results

A total of 69 subjects were enrolled in this study. The healthy group was composed of 32 subjects and the intubation group 37. The age range for all subjects was 22 to 77 years old. The mean age of patients in the healthy and intubation groups was 45. 81±14.26 and 48.72±14.32, respectively (p > 0.05). There were 16 men and 21 women in the intubation group, and the inner diameters of the tubes were 7 mm and 6.5 mm, respectively. A total of 17 patients experience PAST and the incidence in our study was approximately 46%. Among them, 10 (10/21, 47.6%) were female and 7 (7/16, 43.7%) were male and there was no statistically significant relationship between sex and PAST. Meanwhile, in the intubation group, 20 cases were anesthetized for thyroid surgery, and the remaining 17 cases underwent paranasal sinus surgery. The tubes were all fixed in the midline area of the mouth in thyroid cases, while in cases with sinus surgery, all the tubes were fixed to the left corner of the mouth. The incidence of PAST was 45% and 47.1% (9/20 and 8/17) in a patient with thyroid and sinus surgery, respectively. No significant difference in the incidence of PAST between the types of surgery was found (p > 0.05).

**Figure 2:**
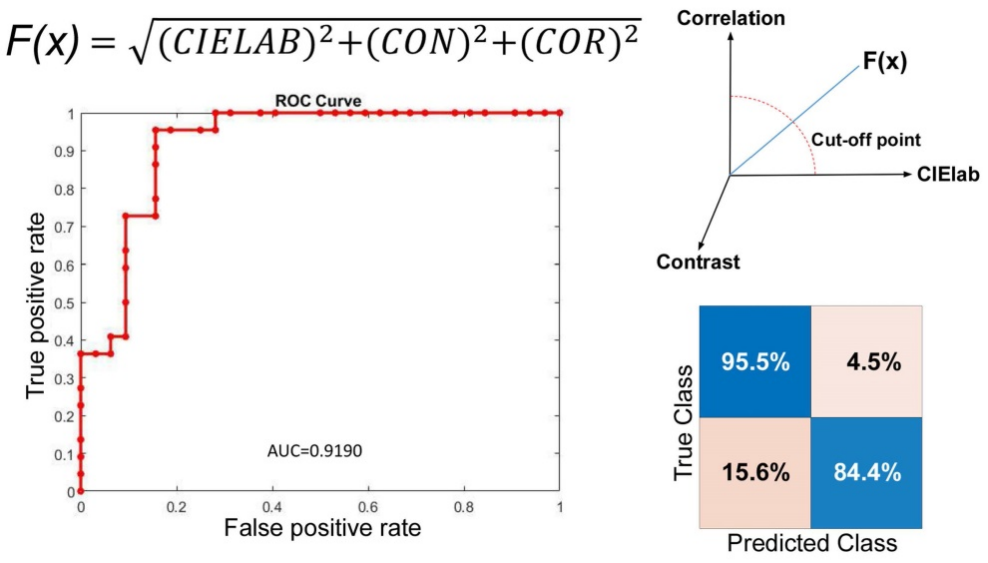
ROC curve of our PAST index to predict post-intubation larygneal inflammation. Our measurement has a sensitivity of 95.5% and a specificity of 84.4%.

Laryngoscopic images were obtained in the healthy group (Fig. 1C) and the intubation group within 24 hours post-surgery (Fig. 1D). The larynx was sub-segmented to the vocal fold and interarytenoid region. The CIELab in interarytenoid region was calculated between two groups. The measures were shown to be significantly higher in the intubation group than in the healthy group, with a p value <0.001. Meanwhile, texture analysis in the interarytenoid region with contrast and correlation profiles also showed similar trends toward higher measures in the intubation group than in the healthy group, with statistical significance (Table 1). Receiver operating characteristic (ROC) curves were used to determine the cutoff values to detect patients with a high risk of postoperative laryngeal inflammation. Based on the results from Figure 2, we yielded an overall classification accuracy of 88.9%, with an area under the ROC curve (AUC) of 0.919. High area under the ROC curve (AUC) values suggested that our PAST index was “outstanding” at classifying a high risk for postoperative laryngeal inflammation (Table 1, Fig. 2). This indicates that our equipment and processing were not only accurate but also has a high measurement of discrimination. This may serve as a valuable tool in a clinical setting, which would allow a clinician to diagnose postoperative laryngeal inflammation in a simpler and less invasive way.

**Figure 3:**
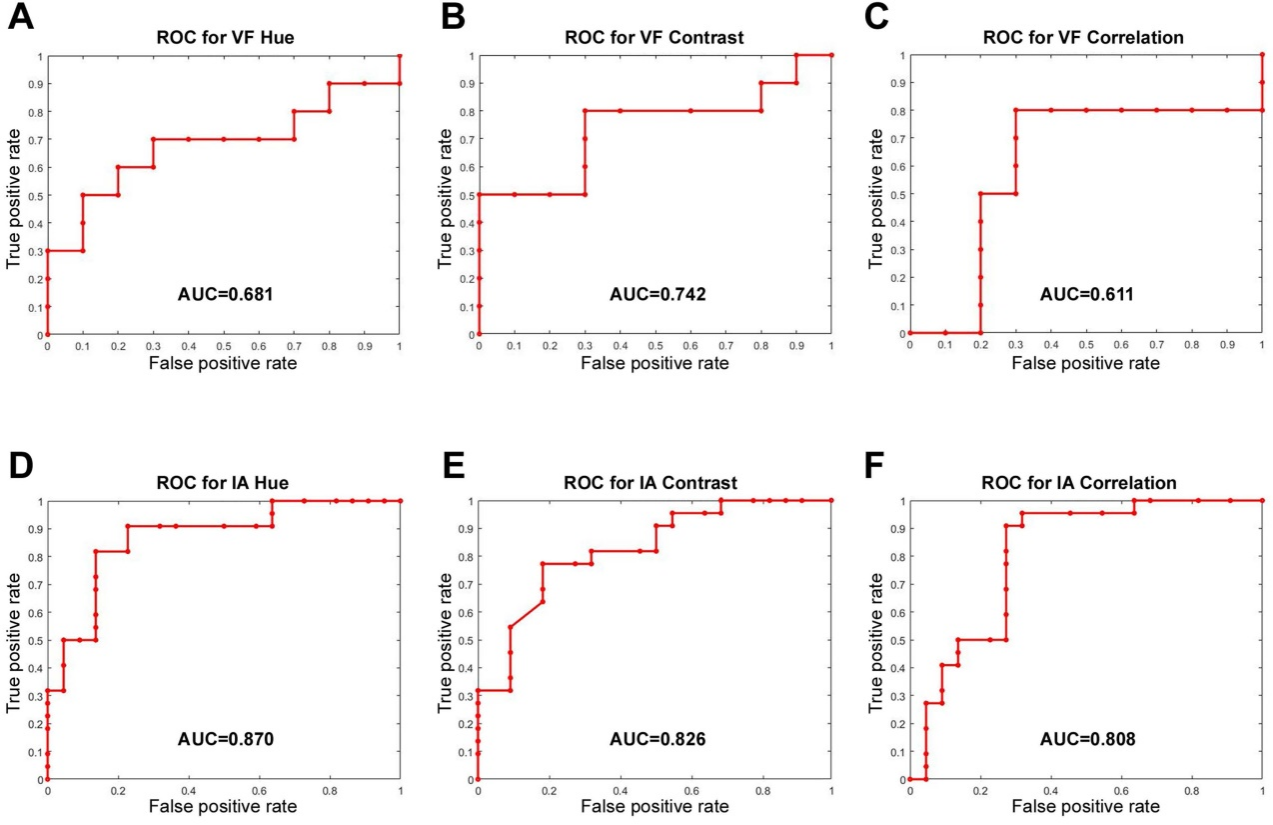
ROC curve analysis of different hue and texture features for vocal fold (VF) **(A, B, C)** and interarytenoid region (IA) **(D, E, F)**. The discrimination was significantly better in IA than in VF.

In the intubation group, images with a noticeable blur or glare were omitted from this study and, finally, 32 subjects with clear pre-and post-surgery images were analyzed. We quantified the average color value, hue and texture features, including contrast, correlation, energy, and homogeneity, to analyze the degree of erythema and inflammation pre and post-surgery, in the true vocal fold and interarytenoid region, respectively. In the true fold region, the AUC of hue, contrast, and correlation were 0.681, 0.742, and 0.611, respectively. While in the interarytenoid region, significantly better discrimination was found and the AUC of hue, contrast, and correlation were 0.870, 0.826, and 0.808, respectively (Fig. 3). This might indicate that the interarytenoid region played a more important role in identifying the postoperative laryngeal inflammation, in terms of color or texture changes. Afterwards, texture analysis was performed according to different hue conditions (R, G, B). Except for energy measure in G color, all the other eigenvalues showed a significant difference before and after surgery under general anesthesia with intubation in the interarytenoid region (Table 2). Spectroradiometric objective color measurements and the CIELab were used together with contrast and correlation profile from texture analysis. All measures showed a statistically significant increases post extubation (all p<0.001) and the PAST index showed outstanding discrimination (Table 3).

The maximum value of the glottic area, vocal fold angle, vocal fold length, width (at midpoint), and area are summarized in Table 4. The glottic area showed a significant decrease of 25.29% post extubation. Similar trends toward smaller vocal angle with 1.15% decreases post extubation, but without statistical significance. Both the decreases in the glottic area and vocal fold angle indicated laryngeal edema that resulted in decreased airway patency. The vocal length revealed no significant change, but both the vocal width and area are significantly increased post general anesthesia. The width increased from 3.01 to 3.57 mm on left, while from 2.88 to 3.42 mm on the right. Although without statistical significance, the vocal width on the right increased a bit more than the one on the left (18.83 VS 18.5%). Our result demonstrated that although color and texture change in the true fold region was obscure, the absolute value of the actual size change still has clinical benefit in identifying postoperative inflammatory changes.

## Discussion

In the past, the description of PAST remained qualitative or semi-quantitative by using a visual analogue scale (VAS) or numeric rating scale (NRS) to measure its intensity, which is often largely subjective. As for post-operative hoarseness, voice assessment with perceptual rating scale was usually carried out by a speech pathologist, which was still based on the examiner's judgment with unignorable inter-observer variance. To our knowledge, this is the first report of a standardized and computer-based quantitative analysis of the post-operative changes in the human larynx. In our previous report 5, we solved some adverse collateral factors of digital evaluation and standardized the measurement of the human larynx with scaling references by using a laser projection module. In this research, we further highlight its clinical application in assessing the sequelae to the vocal cords following anaesthesia with intubation. Our incidence of post-extubation throat complaints of approximately 46% lies in the middle of ranges of comparable figures from the literature (14-75%) 10-11. In the present study variables such as age, gender, type of surgery did not significantly influence the occurrence of PAST and our texture profiles. Previous studies have shown that compared with men, more women have PAST 12. Meanwhile, an increased incidence of PAST following thyroid surgery has been reported 13. Several reasons were proposed, such as airway manipulation was more common during thyroid surgery, or dryness in the mouth and throat were more common in women. However, our study shows that, regardless of gender or thyroid surgery, PAST and hoarseness are a subjective concern for the patient. At least there were no statistically significant changes in the larynx between different characteristics. The source of the additional pain in women or patients post thyroid surgery may not solely come from the laryngeal inflammation. In this case, clinicians should focus on oral cavity or external cervical wounds to alleviate the patient's discomfort.

Our study obtained several interesting results after statistically analyzing the associations between intubation and laryngeal color and texture profiles. The CIELab is a color space modeled on the visual pattern of humans and designed to be perceptually uniform in human perceptual work 14. One advantage is that the differences in lighting could be better controlled by using CIELab, instead of hue. Textural analysis attempts to quantitatively describe characteristics of images based on the spatial arrangement of intensity values 15. It is an image processing algorithm that is based on analyzing the distribution and relationship of pixel or voxel-gray levels in the image, which is only qualitatively assessable by the human visual system to a limited degree 16. The computer-assisted image texture analysis has the potential to allow the extraction of quantitative parameters related to tissue heterogeneity, which has proven to be useful in lung cancer identification 17. In this study, we use CIElab and texture analysis to provide a nuanced, robust distinction solely between laryngeal mucosa affected by intubation and that which is not. Our approach yielded an optimal classification accuracy of 88.9%. The corresponding ROC curve provided an area under the curve equal to 0.919, indicating outstanding sensitivity and specificity. Our quantification allows for objective visualization of the larynx by creating a quantified color and texture profile, independent of subjective clinical observations. Subsequently, we compared the hue and texture profiles over the interarytenoid region before and post-surgery. All measures (contrast, correlation, energy, and homogeneity) show significant distinction pre and post operation, with AUC of 0.807~0.869. As for the true vocal fold region, the analysis showed insufficiency to no discrimination, with AUC around 0.5~0.6. Therefore, the inflammation over the interarytenoid region was more severe than the true vocal fold region. This was consistent with the clinical setting because the endotracheal tube was placed mainly upon the interarytenoid mucosa. Only a thin stratified squamous epithelium and underlying lamina propria comprise the surface of the vocal fold, with sparse cell population and lesser bloody supply than other laryngeal mucosa. Our results also indicated that our measurement is not only accurate but also has a high measurement of discrimination, which would allow a clinician to approach PAST in a simpler and less invasive way. Those who complain about severe PAST might not have objective signs suggestive of inflammation presence, and our work will benefit the clinician who would otherwise make a differential diagnosis based solely on subjective interpretation of nonspecific laryngeal signs.

The postoperative laryngeal complications could be a result of direct or indirect trauma to the vocal cords causing edema, hematoma, or nerve stretch 18. For the first time, our study provided significant direct objective evidence, that intubation will lead to laryngeal inflammation, which decreased the glottic area and vocal angle and increased the vocal fold width and area. An increase in width and area of the vocal folds indicated the vocal fold edema. The increased vocal mass might change the fundamental frequency and disrupt the harmonic to noise ratio, which results in postoperative dysphonia or pitch break. In the past, *in vivo* precise measurement of the laryngeal parameters, such as vocal fold length or width, was very difficult due to lack of suitable tools and poor compliance owing to severe gag reflexes. Previous researches had made various attempts, including estimation from cadavers or plain films 19-20. But none of those could directly reflect the physiological vocal fold condition in living subjects and the inaccurate measurement made precise interpretation of the data difficult. In our previous study, we provided evidence that our approach could give a more precise vocal fold measurement in living subjects. In the present study, the right vocal fold width increased more than the left. We had once discussed this with anesthesiologists and they supposed it was because we usually fixed the tracheal intubation on the right side. But when we look deeper, we found that the fixed position of the tube was irrelevant to our result. Therefore, we hypothesized that the occurrence of this phenomenon came from the process of intubation. Since most anesthesiologists were right-handed, it was reasonable to conclude the right vocal cord sustains more stress and impact during intubation, no matter what technique or device was used (such as traditional or glidescope intubation). Although there was no statistical significance, the wider and heavier right vocal fold might hint that the intubation process played an important role in the formation of postoperative laryngeal trauma and we should improve the insertion technique to avoid sequelae.

To alleviate PAST, previous researches have attempted the application of anti-inflammatory or analgesic medicine on the ETT cuff, such as lidocaine or steroid spray 21. Several studies have shown that lidocaine spray or lubricating jelly might be a cause of PAST due to its additive 22. In our clinical practice, we routinely use benzydamine spray on the ETT cuff. Besides, to use intraoperative nerve monitor, inadequate neuromuscular block in thyroid surgery might be the reason for increased PAST noted in literature 23. Remedial action would be to use a smaller size tube, presumably because of the decreased pressure at the mucosal interface 24. Despite considerable effort and care during intubation, PAST will still occur and earlier studies have not confirmed that the insertion technique is associated with postoperative sequelae 25. Lack of appropriate objective quantitative evaluation tools might be the main reason. As a result, it has been difficult to devise protocols to minimize such injuries. With our new device, again, we can conduct a prospective study to compare different insertion techniques among different surgery, intubating conditions, or variable anesthetic factors. Despite this, our study might have limited contribution to the comprehension of association between throat color and intubation, as we focused our study on the laryngeal region only and did not include other factors such as the oropharyngeal or oral cavity condition, the experience of the intubator, or the use of airway suction. We did not control or standardize the anesthesia protocol, but no persons in training were allowed to intubate in this series. The cohort was small, and the relationship between the severity of PAST and our quantitative measures cannot be analyzed due to limited number of cases. Meanwhile, patients may have shallow breathing during postoperative laryngoscopy, and the parameters we measured may not be perfect. The strength of this study design is that the results reflect a real-life situation. The performance of our predictive models is also fairly good. To increase the validity and generalization of our results, another known determinant of PAST should be considered and this study could be extended by conducting the study on oropharynx or oral cavity mucosa in the future.

## Conclusion

This is the first report of a truly objective morphometric measurement and quantitative analysis of laryngeal structures profiles pre- and post-surgery under general anesthesia with intubation. The trend to red and the increased contrast texture were compatible with post-intubation inflammatory response, especially in the interaryteonid mucosa. Furthermore, wider vocal fold and reduced glottic space suggested voice change or shortness of breath post extubation. However, the occurrence of PAST was not related to the type of surgery or sex in our study. Our equipment and processing may serve as a valuable tool in a clinical setting, which would allow a clinician to diagnose postoperative laryngeal inflammation in a simpler and less invasive way, so as to take appropriate steps to prevent it in the future.

## Figures and Tables

**Table 1:** The eigenvalue of color space and texture feature of patients in the healthy control group and intubation group

	Control group (32)	Intubation group (37)	*p* value
CEILab	48.92±5.53	59.98±4.80	0.000
Contrast *1000	5.33±2.58	9.05±3.45	0.000
Correlation *100	93.62±1.98	95.31±1.54	0.000
PAST index	105.88±3.93	113.09±3.36	0.000

Test applied: Student *t* test.

**Table 2:** The eigenvalue of the texture profile corresponding to the patient's different hue features pre and post intubation (n=32)

	R		G		B	
	Pre	Post	Pre	Post	Pre	Post
Contrast *1000	5.65±2.34	8.16±3.57	3.68±1.56	5.45±2.65	3.03±1.37	4.53±2.42
Energy *100	99.15±0.37	99.42±0.33	98.68±1.90	99.38±0.31	99.16±0.37	99.42±0.33
Correlation *100	92.61±2.61	94.69±1.58	92.87±2.68	94.95±2.10	92.13±2.66	94.35±1.69
Homogeneity *100	99.91±0.04	99.93±0.03	99.91±0.04	99.94±0.03	99.91±0.04	99.94±0.03

**Table 3:** The eigenvalue of the texture profile corresponding to the CEILab in a patient pre and post intubation. (n=32)

	Pre	Post	*p* value
CEILAB	52.63±4.40	58.75±4.02	0.000
Contrast *1000	6.74±2.85	10.37±3.07	0.000
Correlation *100	94.35±1.83	95.87±1.13	0.000
PAST index	108.34±3.30	112.99±2.92	0.000

Test applied: Paired samples t-test.

**Table 4:** Characteristics and the laryngeal measurements of the patients pre and post intubation (n=32)

	Pre	Post	Ratio	*p* value
Area of glottic space (mm^2^)	103.60±11.02	77.40±6.45	-25.29%	0.002
LVL (mm)	14.03±0.48	14.46±0.65	3.03%	0.932
LVW (mm)	3.01±0.09	3.57±0.19	18.50%	0.005
LVA (mm^2^)	26.97±1.61	35.42±3.21	31.33%	0.020
RVL (mm)	14.07±0.50	14.05±0.61	-0.15%	0.717
RVW (mm)	2.88±0.07	3.42±0.14	18.83%	0.000
RVA (mm^2^)	27.06±1.63	35.80±2.67	32.29%	0.004
TVA (mm^2^)	54.03±3.11	71.14±5.79	31.66%	0008
Vocal fold angle (degree)	42.37±2.48	41.89±3.69	-1.15%	0.688

LVL: left vocal length; LVW: left vocal width; LVA: left vocal area; RVL: right vocal length; RVW: right vocal width; RVA: right vocal area; TVA: total vocal area; Test applied: Paired samples t-test.

## References

[1] Kuriyama A, Maeda H, Sun R (2019). Topical application of magnesium to prevent intubation-related sore throat in adult surgical patients: a systematic review and meta-analysis. Can J Anaesth.

[2] Puyo CA, Dahms TE (2012). Innate immunity mediating inflammation secondary to endotracheal intubation. Arch Otolaryngol Head Neck Surg.

[3] Biro P, Seifert B, Pasch T (2005). Complaints of sore throat after tracheal intubation: a prospective evaluation. Eur J Anaesthesiol.

[4] Kwak JT, Xu S, Wood BJ (2015). Efficient Data Mining for Local Binary Pattern in Texture Image Analysis. Expert Syst Appl.

[5] Kuo CJ, Lin CS, Chiang KY, Barman J, Liu SC (2021). *In vivo* Automatic and Quantitative Measurement of Adult Human Larynx and Vocal Fold Images. J Voice.

[6] Kuo CJ, Kao CH, Dlamini S, Liu SC (2020). Laryngopharyngeal reflux image quantization and analysis of its severity. Sci Rep.

[7] Bagchi D, Mandal MC, Das S, Sahoo T, Basu SR, Sarkar S (2012). Efficacy of intravenous dexamethasone to reduce incidence of postoperative sore throat: A prospective randomized controlled trial. J Anaesthesiol Clin Pharmacol.

[8] Lillotte TD, Joester M, Frindt B, Berghaus A, Lammens RF, Wagner KG (2021). UV-VIS spectra as potential process analytical technology (PAT) for measuring the density of compressed materials: Evaluation of the CIELAB color space. Int J Pharm.

[9] Albà X, Lekadir K, Pereañez M, Medrano-Gracia P, Young AA, Frangi AF (2018). Automatic initialization and quality control of large-scale cardiac MRI segmentations. Med Image Anal.

[10] Brodsky MB, Akst LM, Jedlanek E, Pandian V, Blackford B, Price C, Cole G, Mendez-Tellez PA, Hillel AT, Best SR, Levy MJ (2021). Laryngeal Injury and Upper Airway Symptoms After Endotracheal Intubation During Surgery: A Systematic Review and Meta-analysis. Anesth Analg.

[11] Christensen AM, Willemoes-Larsen H, Lundby L, Jakobsen KB (1994). Postoperative throat complaints after tracheal intubation. Br J Anaesth.

[12] Hu B, Bao R, Wang X, Liu S, Tao T, Xie Q, Yu X, Li J, Bo L, Deng X (2013). The size of endotracheal tube and sore throat after surgery: a systematic review and meta-analysis. PLoS One.

[13] Gong Y, Xu X, Wang J, Che L, Wang W, Yi J (2020). Laryngeal mask airway reduces incidence of post-operative sore throat after thyroid surgery compared with endotracheal tube: a single-blinded randomized controlled trial. BMC Anesthesiol.

[14] Altmann CS, Brachmann A, Redies C (2021). Liking of art and the perception of color. J Exp Psychol Hum Percept Perform.

[15] Kockelkorn TT, de Jong PA, Schaefer-Prokop CM, Wittenberg R, Tiehuis AM, Gietema HA, Grutters JC, Viergever MA, van Ginneken B (2016). Semi-automatic classification of textures in thoracic CT scans. Phys Med Biol.

[16] Wang ZZ, Yong JH (2008). Texture analysis and classification with linear regression model based on wavelet transform. IEEE Trans Image Process.

[17] Bashir U, Siddique MM, Mclean E, Goh V, Cook GJ (2016). Imaging Heterogeneity in Lung Cancer: Techniques, Applications, and Challenges. AJR Am J Roentgenol.

[18] Campbell BR, Shinn JR, Kimura KS, Lowery AS, Casey JD, Ely EW, Gelbard A (2020). Unilateral Vocal Fold Immobility After Prolonged Endotracheal Intubation. JAMA Otolaryngol Head Neck Surg.

[19] Hu Q, Zhu SY, Luo F, Gao Y, Yang XY (2010). High-frequency sonographic measurements of true and false vocal cords. J Ultrasound Med.

[20] Schuberth S, Hoppe U, Dollinger M, Lohscheller J, Eysholdt U (2002). High-precision measurement of the vocal fold length and vibratory amplitudes. Laryngoscope.

[21] Kim D, Jeong H, Kwon J, Kang S, Han B, Lee EK, Lee SM, Choi JW (2019). The effect of benzydamine hydrochloride on preventing postoperative sore throat after total thyroidectomy: a randomized-controlled trial. Can J Anaesth.

[22] Soltani HA, Aghadavoudi O (2002). The effect of different lidocaine application methods on postoperative cough and sore throat. J Clin Anesth.

[23] Hisham AN, Roshilla H, Amri N, Aina EN (2001). Post-thyroidectomy sore throat following endotracheal intubation. ANZ J Surg.

[24] Farrow S, Farrow C, Soni N (2012). Size matters: choosing the right tracheal tube. Anaesthesia.

[25] L'Hermite J, Dubout E, Bouvet S, Bracoud LH, Cuvillon P, Coussaye JE, Ripart J (2017). Sore throat following three adult supraglottic airway devices: A randomised controlled trial. Eur J Anaesthesiol.

